# Clinical Characterization and Genomic Analysis of Samples from COVID-19 Breakthrough Infections during the Second Wave among the Various States of India

**DOI:** 10.3390/v13091782

**Published:** 2021-09-07

**Authors:** Nivedita Gupta, Harmanmeet Kaur, Pragya Dhruv Yadav, Labanya Mukhopadhyay, Rima R. Sahay, Abhinendra Kumar, Dimpal A. Nyayanit, Anita M. Shete, Savita Patil, Triparna Majumdar, Salaj Rana, Swati Gupta, Jitendra Narayan, Neetu Vijay, Pradip Barde, Gita Nataraj, Amrutha Kumari B., Manasa P. Kumari, Debasis Biswas, Jyoti Iravane, Sharmila Raut, Shanta Dutta, Sulochana Devi, Purnima Barua, Piyali Gupta, Biswa Borkakoty, Deepjyoti Kalita, Kanwardeep Dhingra, Bashir Fomda, Yash Joshi, Kapil Goyal, Reena John, Ashok Munivenkatappa, Rahul Dhodapkar, Priyanka Pandit, Sarada Devi, Manisha Dudhmal, Deepa Kinariwala, Neeta Khandelwal, Yogendra Kumar Tiwari, Prabhat Kiran Khatri, Anjli Gupta, Himanshu Khatri, Bharti Malhotra, Mythily Nagasundaram, Lalit Dar, Nazira Sheikh, Jayanthi Shastri, Neeraj Aggarwal, Priya Abraham

**Affiliations:** 1 Indian Council of Medical Research, V. Ramalingaswami Bhawan, Ansari Nagar, New Delhi 110029, India; drguptanivedita@gmail.com (N.G.); harmanmeet.kaur@gmail.com (H.K.); labanya.mukhopadhyay@gmail.com (L.M.); salajrana05@gmail.com (S.R.); 07guptaswati@gmail.com (S.G.); jitunarayan@gmail.com (J.N.); drneetuvijay@gmail.com (N.V.); aggarwal.n@icmr.gov.in (N.A.); 2 Indian Council of Medical Research-National Institute of Virology, Pune 411021, India; dr.rima.sahay@gmail.com (R.R.S.); abhinendra.biotech@gmail.com (A.K.); nyayanit.dimpal@gmail.com (D.A.N.); anitaaich2008@gmail.com (A.M.S.); varshapatil111@yahoo.com (S.P.); triparna.majumdar@gmail.com (T.M.); yashjos1401@gmail.com (Y.J.); priyanka.pb83@gmail.com (P.P.); dudhmalmanisha23@gmail.com (M.D.); priya.abraham@icmr.gov.in (P.A.); 3 Viral Research and Diagnostic Laboratory, National Institute of Research in Tribal Health (NIRTH), Jabalpur 482003, India; pradip_barde@hotmail.com; 4 Viral Research and Diagnostic Laboratory, Department of Microbiology, KEM Medical College, Mumbai 400012, India; gitanataraj@gmail.com; 5 Viral Research and Diagnostic Laboratory, Department of Microbiology, Mysore Medical College, Mysore 570015, India; amrutakb@yahoo.co.in (A.K.B.); manasavinay22@gmail.com (M.P.K.); 6 Viral Research and Diagnostic Laboratory, Department of Microbiology, All India Institute of Medical Sciences, Bhopal 462020, India; debasis.microbiology@aiimsbhopal.edu.in; 7 Viral Research and Diagnostic Laboratory, Government Medical College, Aurangabad 431001, India; jairavane@hotmail.com; 8 Viral Research and Diagnostic Laboratory, Indira Gandhi Government Medical College, Nagpur 440012, India; sharmilakuber@gmail.com; 9 Viral Research and Diagnostic Laboratory, National Institute of Cholera and Enteric Diseases, Kolkata 700010, India; drshantadutta@gmail.com; 10 Viral Research and Diagnostic Laboratory, Regional Institute of Medical Sciences, Imphal 795004, India; sulo_khu@rediffmail.com; 11 Viral Research and Diagnostic Laboratory, Jorhat Medical College, Jorhat 785001, India; drpurnimabarua@gmail.com; 12 Viral Research and Diagnostic Laboratory, Mahatma Gandhi Memorial Medical College, Jamshedpur 831020, India; mgmvrdl@gmail.com; 13 Viral Research and Diagnostic Laboratory, ICMR-Regional Medical Research Centre, Dibrugarh 786001, India; biswaborkakoty@gmail.com; 14 Viral Research and Diagnostic Laboratory, All India Institutes of Medical Sciences, Rishikesh 249203, India; deep.micro@aiimsrishikesh.edu.in; 15 Viral Research and Diagnostic Laboratory, Government Medical College, Amritsar 143001, India; kdmicrogmcasr@gmail.com; 16 Viral Research and Diagnostic Laboratory, Sher-i-Kashmir Institute of Medical Sciences, Srinagar 190011, India; bashirfomda@gmail.com; 17 Department of Virology, Postgraduate Institute of Medical Education and Research, Chandigarh 160012, India; kapilgoyalpgi@gmail.com; 18 Viral Research and Diagnostic Laboratory, Government Medical College, Thrissur 680596, India; rejovi3@gmail.com; 19 ICMR-National Institute of Virology Field Unit, Bangalore 560011, India; ashokmphdns@gmail.com; 20 Viral Research and Diagnostic Laboratory, Jawaharlal Institute of Postgraduate Medical Education & Research, Puducherry 605006, India; rahuldhodapkar@gmail.com; 21 Viral Research and Diagnostic Laboratory, Government Medical College, Thiruvanthapuram 695011, India; sdevikl23@gmail.com; 22 Viral Research and Diagnostic Laboratory, B. J. Medical College, Ahmedabad 380016, India; poliobjmedical@gmail.com; 23 Viral Research and Diagnostic Laboratory, Government Medical College, Surat 395001, India; neetashokk@gmail.com; 24 Viral Research and Diagnostic Laboratory, Jhalawar Medical College, Jhalawar 326001, India; yogendratiwari2012@gmail.com; 25 Viral Research and Diagnostic Laboratory, Dr. Sampurnanand Medical College, Jodhpur 342003, India; drpkkhatri@yahoo.co.in; 26 Viral Research and Diagnostic Laboratory, Sarder Patel Medical College, Bikaner 334001, India; vrdlbikaner@gmail.com; 27 Viral Research and Diagnostic Laboratory, Department of Microbiology, GMERS Medical College, Himmatnagar 383001, India; microgmershmt@gmail.com; 28 Viral Research and Diagnostic Laboratory, Sawai Man Singh Medical College, Jaipur 302004, India; drbhartimalhotra@gmail.com; 29 Viral Research and Diagnostic Laboratory, Coimbatore Medical College, Coimbatore 641018, India; mythilynmicro@gmail.com; 30 Viral Research and Diagnostic Laboratory, All India Institute of Medical Sciences, Delhi 110029, India; lalitdaraiims@gmail.com; 31 Viral Research and Diagnostic Laboratory, Dr. V.M Government Medical College, Solapur 413003, India; vrdlsolapur@gmail.com; 32 Viral Research and Diagnostic Laboratory, Kasturba Hospital for Infectious Diseases, Mumbai 400011, India; jsshastri@gmail.com

**Keywords:** breakthrough, COVID-19, VRDL, Delta and Delta plus variant, India, vaccine

## Abstract

From March to June 2021, India experienced a deadly second wave of COVID-19, with an increased number of post-vaccination breakthrough infections reported across the country. To understand the possible reason for these breakthroughs, we collected 677 clinical samples (throat swab/nasal swabs) of individuals from 17 states/Union Territories of the country who had received two doses (*n* = 592) and one dose (*n* = 85) of vaccines and tested positive for COVID-19. These cases were telephonically interviewed and clinical data were analyzed. A total of 511 SARS-CoV-2 genomes were recovered with genome coverage of higher than 98% from both groups. Analysis of both groups determined that 86.69% (*n* = 443) of them belonged to the Delta variant, along with Alpha, Kappa, Delta AY.1, and Delta AY.2. The Delta variant clustered into four distinct sub-lineages. Sub-lineage I had mutations in ORF1ab A1306S, P2046L, P2287S, V2930L, T3255I, T3446A, G5063S, P5401L, and A6319V, and in N G215C; Sub-lineage II had mutations in ORF1ab P309L, A3209V, V3718A, G5063S, P5401L, and ORF7a L116F; Sub-lineage III had mutations in ORF1ab A3209V, V3718A, T3750I, G5063S, and P5401L and in spike A222V; Sub-lineage IV had mutations in ORF1ab P309L, D2980N, and F3138S and spike K77T. This study indicates that majority of the breakthrough COVID-19 clinical cases were infected with the Delta variant, and only 9.8% cases required hospitalization, while fatality was observed in only 0.4% cases. This clearly suggests that the vaccination does provide reduction in hospital admission and mortality.

## 1. Introduction

Severe acute respiratory syndrome coronavirus-2 (SARS-CoV-2) was reported from Wuhan, China, in December 2019 and rapidly spread across the globe. The World Health Organization (WHO) declared the disease caused by it, Coronavirus disease of 2019 (COVID-19) as a Public Health Emergency of International Concern on 11t March 2020. Since then, the virus has been continuously evolving, and the first major mutation was seen in its spike protein (D614G), which led to increased infectivity [1]. However, several new SARS-CoV-2 variants of concern (VOCs), i.e., Alpha (B.1.1.7), Beta (B.1.351), and Gamma (B.1.1.28.1), have been detected from United Kingdom, South Africa, and Brazil, respectively, from September to December 2020 and have also been reported from India [2,3,4]. Our earlier study of genomic surveillance from January to August 2020 showed the absence of VOC/variants under investigation (VUIs) and the presence of the G, GR, and GH clade in the country, with a number of mutations [5]. The global circulation of variants amplified the COVID-19 pandemic with increased transmissibility, enhanced severity of illness, diminished protection relative to previous SARS-CoV-2 variant infection, and lower response to vaccines and monoclonal antibodies [6,7,8].

Since the worldwide alert of VOCs, international travelers arriving at Indian airports from December 2020 to date were tracked and subjected to diagnostic testing by SARS-CoV-2 specific real-time reverse transcription-polymerase chain reaction (rRTPCR). Genomic surveillance led to the detection of VOCs, i.e., Alpha and Beta; variants of interest (VOIs), i.e., Eta (B.1.525), Kappa (B.1.617.1), and Zeta (B.1.1.28.2); and variant under monitoring, i.e., B.1.617.3 [2,3,4,5,9,10]. The recent emergence of the B.1.617 lineage has created a grave public health problem in India. The lineage evolved further to generate sub-lineages B.1.617.1, B.1.617.2, and B.1.617.3 [11]. The sub-lineage B.1.617.2 has gradually dominated the other variants, including B.1.617.1, B.617.3, and Alpha VOC in Maharashtra state [9,10]. This variant has further evolved into two new strains called Delta AY.1 and Delta AY.2. The AY.1 and AY.2 variants have been aggregated with Delta variant B.1.617.2 [12].

**Figure 1:**
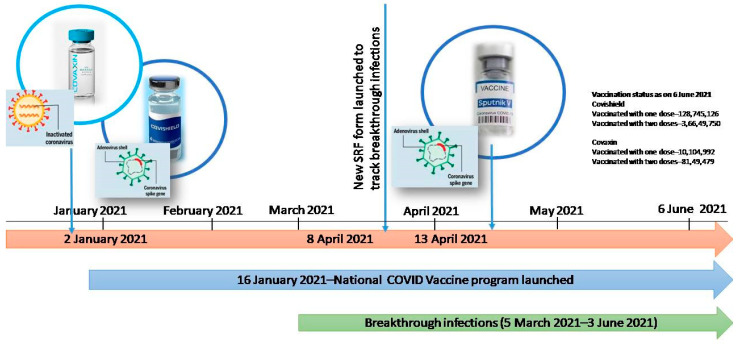
Graphical representation of timelines of vaccine emergency use authorization in India.

Several candidate vaccines have been developed using various platforms on fast-track mode. Many of them have been used in different countries across the globe under emergency use authorization (EUA). On 1 January and 2 January 2021, the National Regulatory Authority of India accorded restricted emergency use authorization to the viral vector vaccine developed by Oxford–AstraZeneca (Covishield, manufactured in India) and inactivated vaccine BBV152 (Covaxin), respectively. Subsequently Sputnik V received EUA on 13 April 2021. In India, the national COVID-19 vaccination program was launched on 16 January 2021. Up to 3 June 2021, 132,847,680 individuals had received one dose, while 45,623,351 individuals had received two doses [13]. The timeline for COVID-19 vaccination in India is graphically represented in Figure 1.

Protection offered by vaccines is being questioned following the emergence of VOCs and reduced real-world effectiveness of certain candidate vaccines against these variants. Israel reported breakthrough COVID-19 infections in individuals immunized with the Pfizer vaccine on 9 April 2021 [14]. In India, we noted a second surge in the number of COVID-19 cases from March 2021, and this was followed by a devastating second wave. Further in April 2021, reduction in neutralization capacity of Covishield/AstraZeneca vaccinated sera against the B.1.1.7 variant as compared with the ancestral strain was observed during in vitro studies [15,16]. Following this, in mid-April 2021, we decided to track breakthrough COVID-19 infections across the country and gain cognizance of the different variants that were responsible for such infections.

In April 2021, Hacisuleyman et al. reported 417 cases of breakthrough infections in individuals vaccinated with Pfizer and Moderna messenger ribonucleic RNA (mRNA) vaccines [17]. Further, post-vaccination breakthrough COVID-19 infections are being reported from all over the globe. The Centers for Disease Control and Prevention (CDC) reported a total of 10,262 COVID-19 vaccine breakthrough infections till April 2021 [18]. Breakthrough infections in healthcare workers vaccinated with the BNT162b2 vaccine were reported from Italy in the May 2021 during an outbreak of SARS-CoV-2 lineage B.1.1.7 [19]. In India, a few studies reported breakthrough infections in small parts of the country, such as in Kerala [20], Chennai [21], and Delhi [22]. Taking cognizance of such reports, in April–May 2021, a nationwide study was undertaken to understand the clinico-demographic profile of patients and SARS-CoV-2 strains responsible for post-vaccination breakthrough COVID-19 infections across the country. To our understanding, this is the largest and first nationwide study of post-vaccination breakthrough infections in India.

## 2. Materials and Methods

### 2.1. Definition of COVID-19 Breakthrough Infection

A breakthrough COVID-19 infection was defined as an individual testing positive for SARS-CoV-2 by rRT-PCR or rapid antigen test (RAT) any time after 14 days of receiving one dose of any of the licensed COVID-19 vaccines.

### 2.2. Study Catchment Area

The Indian Council of Medical Research’s Department of Health Research (ICMR-DHR) utilized a network of viral research and diagnostic laboratories (VRDLs) to track breakthrough infections. Clinical and demographic details as well as nasopharyngeal/oropharyngeal swabs (NPS/OPS) of COVID-19 patients satisfying the case definition were collected by the VRDLs in the north, south, west, east, northeast, and central parts of India from 17 states and Union Territories (UTs) (Maharashtra, Kerala, Gujarat, Uttarakhand, Karnataka, Manipur, Assam, Jammu and Kashmir, Chandigarh, Rajasthan, Madhya Pradesh, Punjab, Pondicherry, New Delhi, West Bengal, Tamil Nadu, and New Delhi) from 5 March 2021 till 3 June 2021. These clinical specimens were sequenced using next-generation sequencing (NGS) to determine nucleotide variations in the SARS-CoV-2 genome from the identified viral strains.

### 2.3. Inclusion, Exclusion Criteria and Transport of Specimens

Cases fulfilling the case definition and the following inclusion criteria were enrolled in the study: (i) cases with or without previous history of COVID-19; (ii) cases whose real-time RT-PCR threshold value was <30 and NPS/OPS were appropriately stored at −80 °C; and (iii) sample referral forms (SRF) capturing the demographic and clinical details of cases were available with the respective VRDLs. All the specimens of breakthrough cases fulfilling the above criteria were packed in triple-layer packaging with dry ice as per International Air Transport Association (IATA) guidelines and then transferred to the reference laboratory at the ICMR-National Institute of Virology (ICMR-NIV), Pune, for sequencing and variant analysis. As depicted in Figure 1, on 8 April 2021, a new specimen referral form (SRF) that included details of COVID-19 vaccination was launched by ICMR throughout the country to capture information related to vaccination status at the time of COVID-19 testing. However, quite a few states had not implemented this new SRF. Therefore, it was not possible to track COVID-19 breakthrough infections in these states, and they were not included in this study.

### 2.4. Retrieval of Clinical and Demographic Data

Though completely filled SRFs were requested along with the specimens, most of the forms received from laboratories were incomplete due to the increased burden of testing during the second wave of COVID-19 in India. Therefore, telephonic interviews were conducted, wherein each reported breakthrough case was called and interviewed individually during the period of 25 May to 14 June 2021. The telephonic interviews also helped in validating the information available in the SRF and in filling in missing data. The patients were questioned on their demographic details, vaccination status, history of earlier COVID-19 infection, contact with a laboratory-confirmed case of COVID-19 prior to breakthrough infection, presence of comorbidities, symptoms developed, and course of infection, including the details of hospitalization. Each phone call typically lasted for 10–12 min, and only patients who provided a complete history were included in the study. A total of 814 clinical specimens were received from the different VRDLs all over the country at ICMR-NIV, Pune. The onset date/OPS and NPS collection dates ranged from 5 March to 3 June 2021, which coincided with the second wave of the COVID-19 pandemic in India. Out of these, 15 patients were not vaccinated for COVID-19, while 2 patients did not give any vaccination history, and 120 patients could not be traced. Thus, after excluding these 137 patients, a total of 677 cases were included in the study. A total of 10 of these 677 patients had documented COVID-19 infection between 8 and 14 days after receiving one dose of vaccine. Though these 10 cases did not satisfy the case definition, they were included in the study, as we did not want to lose any opportunity to detect the newly identified SARS-CoV-2 variants AY.1 and AY.2.

### 2.5. RNA Extraction and Next Generation Sequencing

Total RNA was extracted from 200 to 400 μL of NPS/OPS samples using an automated RNA extraction system (Thermo Fisher, Waltham, MA, USA) using Magmax RNA extraction kit (Applied Biosystems, Waltham, MA, USA). Real time RT-PCR (reverse transcriptase polymerase chain reaction) was set using SARS-CoV-2-specific primers for the detection of E gene and RdRP (RNA-dependent RNA polymerase) gene as described earlier [23]. The IlluminaCovidseq protocol (IlluminaInc, San Diego, CA, USA) was followed for preparation of RNA libraries. Extracted RNA was annealed using random hexamers to prepare for cDNA (complementary DNA) synthesis. The first strand of cDNA was synthesized using reverse transcription. The synthesized cDNA was amplified in two separate PCR plates using two pools of primers (pool 1 and pool 2) covering the entire genome of SARS-CoV-2. Amplified cDNA was then tagmented and bead-based post-tagmentation clean-up was performed. Tagmented amplicons were further amplified in this step using a PCR program as per manufacturer’s instructions (Covidseq reference guide, Illumina). This PCR step added pre-paired 10 base pair indexes (Set 1, 2, 3, 4 adapters), required for sequencing cluster generation. One Covidseq positive control (CPC) and one negative template control (NTC) were used for each 96-well plate. Libraries generated in batches of 96 samples per plate were pooled into one 1.7 mL tube. Libraries of optimal size were purified by using a magnetic bead-based cleanup process method. Amplified and purified libraries were quantified using a KAPA Library Quantification Kit (KapaBiosystems, Roche Diagnostics Corporation, Indianapolis, IN, USA).

For a set of 384 samples, 25 µL of each normalized pool containing index adapter set 1, 2, 3, 4 was combined in a new micro-centrifuge tube. At this step, a final pool of 384 samples was diluted to a starting concentration of 4 nM. These libraries were then denatured, diluted, and then loaded at a final loading concentration of 1.4pM onto the NextSeq 500/550 system using NextSeq 500/550 High Output Kit v2.5 (75 Cycles) as per the manufacturer’s instructions (Illumina Inc., San Diego, CA, USA). The files were analyzed using the reference-based mapping method, as implemented in CLC genomics workbench version 20.0 (CLC, QIAGEN, Aarhus, Denmark). The Wuhan Hu-1 isolate (Accession Number: NC_045512.2) was used as the reference sequence to retrieve the genomic sequence of the SARS-CoV-2. The retrieved sequences were aligned, along with few representative sequences from the GISAID database, in CLC Genomics Workbench v.20. A phylogenetic tree was generated using the MEGA software [24] for the aligned sequences. Gene-wise amino acid mutations were also observed.

## 3. Results

### 3.1. Clinical and Demographic Analysis of the Breakthrough Samples

Detailed distribution of breakthrough cases (*n* = 677) collected from 17 states/UT of the country used for NGS is provided in Table 1. The clinical samples for analysis were collected between March and June 2021. Out of these 677 patients, 85 acquired COVID-19 after taking the first dose of the vaccine, while 592 were infected after receiving both doses of the vaccine. A total of 517 of these 592 individuals contracted COVID-19 after 2 weeks of receiving the second dose of vaccine.

Clinical samples from the COVID-19 cases post second dose of vaccination were collected with a median of 38 days and had an inter-quartile range (IQR) of 20 (19–58) days. A total of 604 patients had received Covishield/AstraZeneca vaccine, 71 had received Covaxin, and 2 had received Sinopharm vaccine (BBIBP-CorV).

Clinical data were analyzed for 677 breakthrough cases. The median age (and the IQR) of patients in the study was 44 (31–56); of the breakthrough cases after one dose, the median age was 53 (45–61), and after two doses it was 41 (30–55). A total of 441 (65.1%) of the breakthrough cases were males. A total of 482 cases (71%) were symptomatic with one or more symptoms, while 29% had asymptomatic SARS-CoV-2 infection. Fever (69%) was the most consistent presentation, followed by body ache, including headache and nausea (56%), cough (45%), sore throat (37%), loss of smell and taste (22%), diarrhea (6%), and breathlessness (6%), and 1% had ocular irritation and redness. The clinical and demographic analysis of the 677 cases of breakthrough infections is enumerated in Table 2.

**Figure 2:**
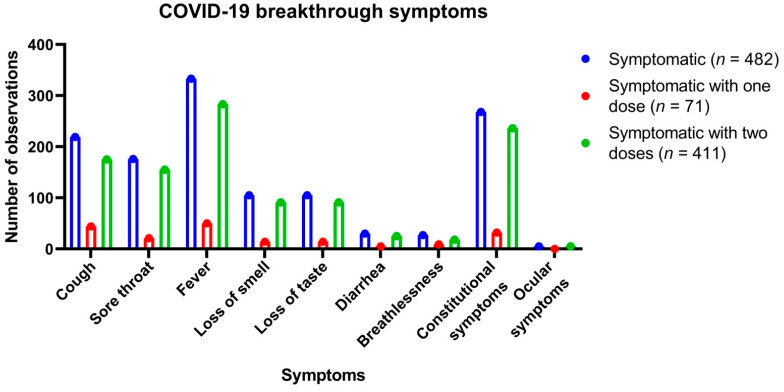
Symptoms reported in COVID-19 breakthrough infections.

Comorbidities were observed in the 154 out of 677 cases, which included diabetes mellitus type 2 and hypertension as well as chronic cardiac, renal, and pulmonary diseases and obesity. The symptoms reported in patients with breakthrough infections are enumerated in Figure 2. The cases with comorbidities were significantly predisposed to develop symptoms (cough, sore throat, fever, loss of smell and taste, diarrhea, breathlessness, ocular symptoms, and constitutional symptoms (body ache, headache, nausea)); (OR = 2.0042, 95% C.I. = 1.2857 to 3.1244, *z*-statistic = 3.069, *p* = 0.0021). Additionally, the cases with medical comorbidities were significantly more predisposed to hospitalization (OR = 3.1779, 95% C.I. = 1.8886 to 5.3471, *z*-statistic = 4.355, *p* < 0.0001).

### 3.2. Vaccine Breakthrough Infections in Individuals with Previous History of COVID-19

Twelve vaccinated individuals gave definitive history of previous laboratory confirmed COVID-19 infection. All of them subsequently received two doses of Covishield/AstraZeneca. The Indian Council of Medical Research had earlier conducted a study that defined re-infection as positive test for SARS-CoV-2 on two separate occasions by either molecular or rapid antigen test at an interval of at least 102 days, with one negative molecular test in between [25]. While we could not elicit history of a negative test result following the first episode of COVID-19 infection, the gap between two positive tests was well above 102 days in 11 cases. One individual received his first dose of COVID-19 vaccine 40 days after testing positive for COVID-19. He received dose two of Covishield/AstraZeneca after 5 weeks, and tested positive 15 days after receiving the second dose. Though the time period between him testing positive twice for SARS-CoV-2 was less than 102 days (89 days), this case was included in the analysis because, to the best of our knowledge, the literature shows that the maximal duration of SARS-CoV-2 shedding that is detectable by PCR is 63 days after onset of symptoms [26]. Hence, we considered this case as a true re-infection and not mere shedding of genomic RNA. This individual was asymptomatic. Median duration from first bout of infection to first dose of vaccination in these 12 cases was 135 days (IQR = 85–166.75 days). Median gap between two doses of the vaccine was 32 days (IQR = 29.75–38 days). Median duration of breakthrough infection from the second dose of the vaccine was 45 days (IQR = 17–55.5 days) and between earlier COVID-19 infection (day of testing) and breakthrough COVID-19 infection (day of testing) was 196 days (IQR = 177.5–249.25 days). Six of these individuals were symptomatic. Most commonly reported symptoms were body ache (4/6), fever (3/6) cough (2/6), sore throat (2/6), headache (2/6), chest pain (1/6). A total of 4 patients had comorbidities, and 1 person out of 12 required hospital admission. He was symptomatic (cough, cold, fever) but had no associated comorbidities.

### 3.3. Next-Generation Sequencing Analysis of the Breakthrough Specimens

Out of 677 cases included in this study, sequencing was not performed for 112 clinical samples (two doses: *n* = 95; single dose: *n* = 17) based on the higher Ct and low Kappa value.

The complete genome of 511 SARS-CoV-2 were recovered with genome coverage of more than 98% (two doses: *n* = 446; single dose: *n* = 65). SARS-CoV-2 sequences with more than 99% and 84% of the genome coverage were recovered from 446 (two doses: *n* = 387; single dose: *n* = 59) and 546 (two doses: *n* = 480; single dose: *n* = 66) clinical specimens, respectively. Less than 98% of genomes were retrieved from 54 samples and were not used further in analysis. The details of the percentage genome retrieved, total reads mapped, and percentage relevant reads are given in Supplementary Table S1. The lineages were retrieved using the Pangolin online software (https://cov-lineages.org/pangolin.html; accessed on 8 August 2021) from the specimens with more than 84% genome coverage and mentioned in Supplementary Table S1.

**Figure 3:**
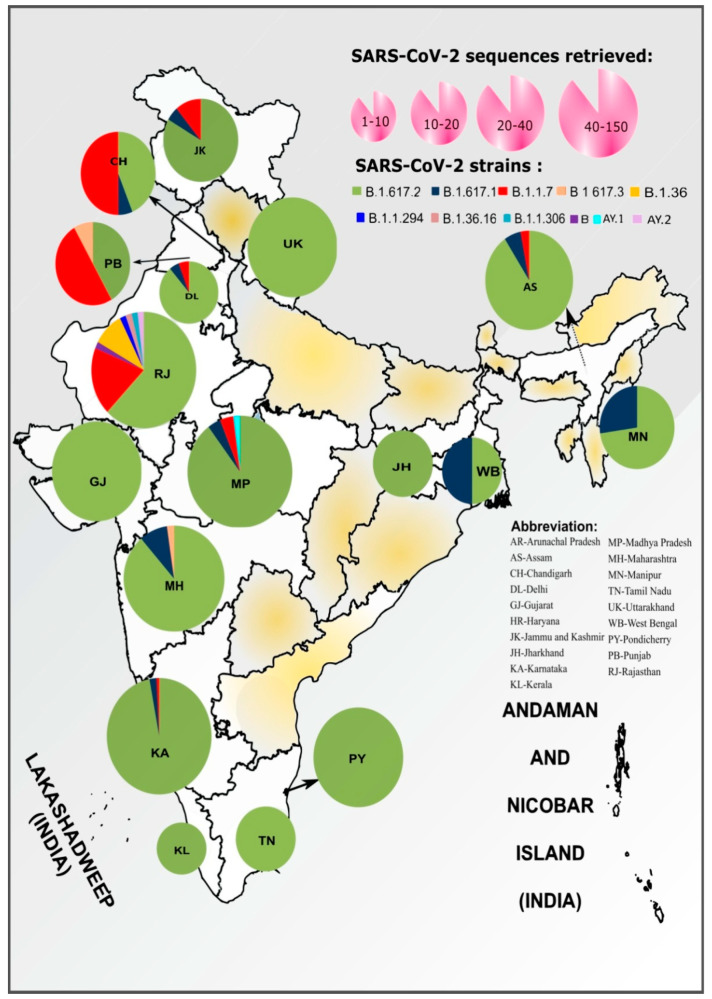
Distribution of the SARS-CoV-2 genome prevalence among cases of breakthrough infection. The size of each pie chart within the states of the India map is ranged based on the number of sequences retrieved in the study. The distribution in the pie chart is proportional to the numbers in each respective clade in each state. The outline of India’s map was downloaded from http://www.surveyofindia.gov.in/file/Map%20f%20India_1.jpg (accessed on 20 March 2020) and further modified to include relevant data in the SVG editor.

The geographic distribution of the different SARS-CoV-2 variants with 98% genome coverage were characterized using Pangolin software and are presented in Figure 3. Delta (B.1.617.2) (*n* = 384) was the major SARS-CoV-2 lineage observed in the breakthrough samples after two doses of vaccine, followed by alpha (B.1.1.7) (*n* = 28). Kappa (B.1.617.1) (*n* = 22), B.1.617.3 (*n* = 2), B (*n* = 1), B.1.36 (*n* = 5), B.1.1.294 (*n* = 1), B.1.36.16 (*n* = 1), B.1.1.306 (*n* = 1), and Delta AY.2 (*n* = 1) pangolin lineage variants were also observed along with others; details are given in Supplementary Table S1. A total of 65 out of 85 samples from individuals infected with SARS-CoV-2 after one dose of vaccination had 99.5% genome retrieval. These sequences had Delta (B.1.617.2) (*n* = 59), Alpha (B.1.1.7) (*n* = 4) Kappa (B.1.617.1) (*n* = 1), and Delta AY.1 (*n* = 1). The Delta AY.1 variant was observed in Madhya Pradesh (MP), while Delta AY.2 was observed in the Rajasthan state of India. The percentage nucleotide divergence of the different SARS-CoV-2 strains relative to reference was 99.81–100%; details for each strain are given in Supplementary Table S1.

It was observed that southern, western, eastern and northwestern regions of India predominantly reported breakthrough infections from mainly Delta and then Kappa variant of SARS-CoV-2. The northern and central regions reported such infections due to Alpha, Delta, and Kappa variants; however, cases due to Alpha variant predominated in the northern region (Figure 3). The overall majority (86.09%) of the breakthrough infections were caused by the Delta variant (B.1.617.2) of SARS-CoV-2 in different regions of India, except for the northern region where the Alpha variant predominated.

Of the 12 cases of breakthrough infection with previous history of COVID-19, 6 samples could be sequenced. These sequences included Delta (B.1.617.2) (*n* = 1), B.1.1.7 (*n* = 2), Kappa (B.1.617.1) (*n* = 1), and B.1.36 (*n* = 2). B.1.1.7 was sequenced from the individual who tested positive for SARS-CoV-2 twice at an interval of 89 days.

**Figure 4:**
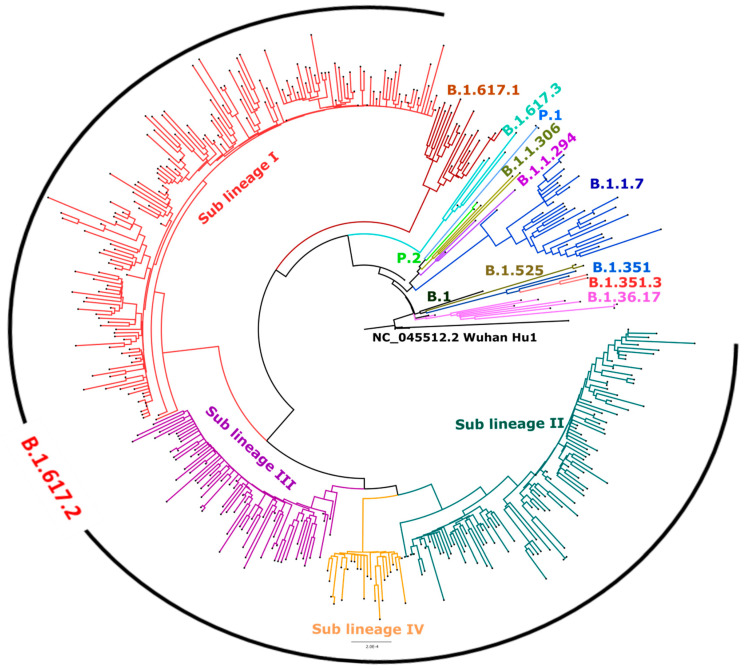
Phylogenetic tree of the 402 SARS-CoV-2 genomes from breakthrough cases with one and two doses vaccine recipients. A Neighbor-joining tree of the 402 SARS-CoV-2 sequences retrieved in this study, along with the representative SARS-Cov-2 sequences from different clades with a bootstrap replication of 1000 cycles. Four major sub-lineages of Delta variant were observed, which are marked on branched in different colors. Sub-lineages I–IV are marked in red, green, pink, and orange color on the nodes, respectively. B.1.617.1 sequence is marked in brown and B.1.617.3 in blue color. The representative pangolin lineages are also marked on branches in different colors. FigTree v1.4.4 and Inkscape were used to visualize and edit the generated tree.

**Figure 5:**
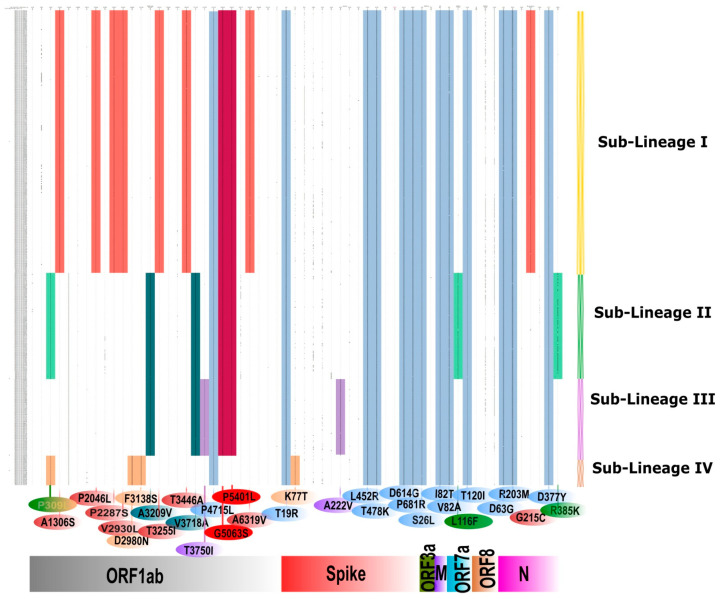
Characterization of sub-lineages observed in the Delta SARS-CoV-2 variant from breakthrough sequences: Sub-lineages I–IV are marked in red, green, pink, and orange color along *y* axis. The amino acid mutations observed in different genes are marked on the *x*-axis. The amino acid change is marked concerning Wuhan-HU-1 (Accession No: NC_045512.2). It is observed that a couple of mutations are conserved in sub-lineages I-III and marked as the gradient of red-violet color. Amino acid mutations conserved for sub-lineage II and III are marked as a violet-green gradient. The amino acid changes common to Delta variant is marked in blue color.

Figure 4 depicts the neighbor-joining tree generated using the Tamura-3-parameter model with a bootstrap replication of 1000 cycles. SARS-CoV-2 sequences (*n* = 421) with genome coverage of 99% and fewer gaps in coding regions were taken for the generation of a phylogenetic tree. A total of 32 representative and 421 SARS-CoV-2 sequences retrieved in this study were used to generate the phylogenetic tree. The Delta sequences (*n* = 358) represented the highest proportion of breakthrough cases from different parts of the country and clustered into four distinct sub-lineages. Sub-lineage I had 166 SARS-CoV-2 sequences, while sub-lineages II, III, and IV had 100, 68, and 24 sequences, respectively, which are marked on the phylogenetic tree. The gene-wise amino acid mutations were further looked upon for the retrieved sequences and the representative sequences relative to the reference sequence. It was observed that the Delta SARS-CoV-2 variant sequences had conservation in different gene positions, leading to differential clustering. These conserved mutations of different sub-lineages are depicted in Figure 5. Sub-lineage I (red color): mutations in ORF1ab A1306S, P2046L, P2287S, V2930L, T3255I, T3446A, G5063S, P5401L, and A6319V and in N G215C; **Sub-lineage II (green color):** ORF1ab P309L, A3209V, V3718A, G5063S, and P5401L and ORF7a L116F; **Sub-lineage III (pink color):** ORF1ab A3209V, V3718A, T3750I, G5063S, and P5401L and spike A222V; **Sub-lineage IV (Orange color):** ORF1ab P309L, D2980N, and F3138S and spike K77T. **Common in B.1.617.2 lineage:** ORF1ab P4715L; spikeT19R, L452R, T478K, D614G, and P681R; ORF3a S26L; M I82T; ORF7a V82A and T120I; and N D63G, R203M, and D377Y.

## 4. Discussion

Globally, COVID-19 vaccines were accorded Emergency Use Authorization (EUA) and introduced quickly into public health programs to prevent SARS-CoV-2 infections and curtail disease transmission, thus saving lives and livelihood. However, the emergence of SARS-CoV-2 VOCs has raised a public health concern due to increased transmissibility and potential to evade humoral immune response. The fact that this has happened amid vaccination uptake has created a dilemma about the efficacy of vaccines under EUA. Data show that there is a 3-fold and 16-fold reduction in neutralization against the Delta and Beta variants as compared with the Alpha variant with BNT162b2 vaccinated sera, and a 5-fold and 9-fold reduction against the same with ChAdOx1 nCoV-19 [27].

As per the WHO classification, the Delta variant has been designated as a variant of concern due to increased transmission and higher immune evasion, whereas the other two sub-lineages of B.1.617—namely, B.1.617.1 and B.1.617.3—with E484Q are grouped in VUI [28]. The B.1.617 variant and its lineage B.1.617.2 were primarily responsible for the surge in COVID-19 cases in Maharashtra state [29]. Delta (B.1.617.2) and Kappa (B.1.617.1) were detected among 60% of the clinical specimens of the COVID-19 cases collected from Maharashtra during January and February 2021 [30]. The rapid rise in daily infections was observed in India, with dominance of the Delta variant, which accounted for >99% of all sequenced genomes in April 2021 [31].A recent study on the secondary attack rates in UK households demonstrated a higher transmission of Delta compared with the Alpha variant [8]. The reduced neutralizing capability of currently used SARS-CoV-2 vaccines against Delta variants is one of the causes for recent increases in breakthrough cases with this strain.

Emergence of VOCs has led to an upsurge in COVID-19 cases and a subsequent wave of pandemic in various countries including India. Incidentally, several countries have reported COVID-19 breakthrough infections even after completion of full vaccination schedules [17,19,30]. More than 10,000 breakthrough infections after completion of a full course of vaccination have been reported in the USA. Overall breakthrough infections were seen in a smaller percentage of the total vaccinated population [18]. A recent study has also reported mild symptomatic breakthrough infections from Kerala and Delhi, India [20,22].

The present study revealed that the infection among breakthrough cases predominantly occurred through the Delta variant, indicating its high community transmission during this period, followed by Alpha and Kappa variants. In our study, 67 cases (9.8%) required hospitalization, and fatality was observed in only 3 cases (0.4%). This clearly suggests that vaccination reduces the severity of disease, hospitalization, and mortality. Therefore, enhancing the vaccination drive and immunizing populations quickly would be the most important strategy for preventing further deadly waves of COVID-19 and would reduce the burden on the health care system.

When COVID-19 vaccination was launched in India on 16 January 2021, the recommended gap between two doses of the Covishield/AstraZeneca vaccine was 4 weeks [32]. Later on, based on studies from the Oxford Vaccine Group and the WHO interim recommendations, the dose interval was increased to 6–8 weeks [33,34] and then to 12–16 weeks within a small time frame. This was based on effectiveness data from the UK [35] and recommendations of Canada [36]. Since Covishield/AstraZeneca contributes to almost 90% of the vaccination in India, increased dose spacing has led to vaccination of greater numbers of eligible people with at least one vaccine dose. However, to tackle the Delta variant of SARS-CoV-2, the UK’s Joint Committee on Vaccination and Immunisation (JCVI) recommended a shortening of the dosing interval to 8 weeks for priority cohorts who are at risk of COVID-19 [37]. Since the Delta variant was predominantly sequenced in our breakthrough infection cases during the second wave of COVID-19 in India, focused studies are now being commissioned in India to look at the need for reducing the gap between two doses of Covishield/AstraZeneca for specific population groups such as immunocompromised individuals, transplant recipients, cancer patients, and people living with HIV.

Two new SARS-CoV-2 variants, Delta AY.1 and AY.2, were also identified in these study samples. The AY.1 and AY.2 variants have been aggregated with Delta variant B.1.617.2 [12]. Delta AY.1 and AY.2 are characterized by the presence of the K417N mutation in the spike protein region. K417N, E484K, L452R, and E484Q are the mutations known to disrupt receptor-binding domain (RBD) binding capacity, making them more infectious by immune escape against the current vaccines [38]. This indicates improved viral fitness to evade immune responses and survive against the vaccines.

Post-vaccination breakthrough COVID-19 cases have been reported from various countries with the use of different licensed vaccines. It appears that the current COVID-19 vaccines are disease-modifying in nature, wherein mild or less severe infections are expected to occur in vaccinated individuals. However, vaccination seems to have an obvious advantage in averting severe disease, hospitalizations, and deaths. Therefore, continuous monitoring of post-vaccination breakthrough infections, along with monitoring of clinical severity of disease, must be adopted as an essential component of vaccine roll-out programs in all countries. Such monitoring will help us to understand the need for adequately tweaking the available vaccines and also for developing new vaccines with enhanced potential to protect against variant strains of SARS-CoV-2.

Identification of the new variants that is responsible for the breakthrough infections underline the importance of this study. It also highlights the need for active genomic surveillance of the new SARS-CoV-2 variants and for assessing their potential to evade immune responses.

## Figures and Tables

**Table 1:** Region-wise and state-wise distribution of SARS-CoV-2 clinical samples used for next-generation sequencing (*n* = 677).

Region	State/UTs	Clinical Samples Received from Each Site
North India	New Delhi	20
	Uttarakhand	50
	Jammu and Kashmir	25
	Punjab	12
	Chandigarh	19
Northeastern India	Manipur	15
	Assam	40
Eastern India	West Bengal	10
	Jharkhand	12
Central India	Madhya Pradesh	68
Western India	Maharashtra	53
	Gujarat	47
	Rajasthan	58
South India	Karnataka	181
	Kerala	16
	Tamil Nadu	25
	Puducherry	26

**Table 2:** Demographic analysis of breakthrough COVID-19 infections.

Characteristics	Vaccinated with Both Doses *	Vaccinated with One Dose *	Total Cases
	(*N* = 592)	(*N* = 85)	(*N* = 677)
	*n* (% of Total)	*n* (% of Total)	*n* (% of Total)
**Age (Years)**			
Median (Interquartile range)	41(30–55)	53 (45–61)	44 (31–56)
**Gender**			
Male	383 (64.7)	58 (68.2)	441 (65.1)
Female	209 (35.3)	27 (31.8)	236 (34.9)
Other	NIL	NIL	NIL
**Comorbidities**			
Yes	134 (22.6)	20 (23.5)	154 (22.7)
No	458 (77.4)	65 (76.5)	523 (77.3)
Missing	NIL	NIL	NIL
**Type of Vaccine**			
Covaxin	63 (10.64)	8 (9.4)	71 (10.5)
**Covishield/AstraZeneca**	527 (89.02)	77 (90.6)	604 (89.2)
**Sinopharm**	2 (0.33)	0	2 (0.3)
**Contact with lab-confirmed SARS-CoV-2 case**	282 (47.6)	31 (36.5)	313 (46.2)
**Median gap between 2 doses of the vaccine(days)**	33(29–41)	NA	NA
**Median (IQR) interval in days between vaccination and SARS-CoV-2 test**	39(19–58)	26(18–38)	NA
**Symptoms during the course of illness**			
**Yes**	411 (69.4)	71 (83.5)	482 (71.2)
**No**	181 (30.6)	14 (16.5)	195 (28.8)
**Hospitalized**	53 (8.9)	14 (16.5)	67 (9.9)
**Individuals with comorbidities ^#^**	22 (3.7)	8 (9.4)	30 (4.4)
**Individuals without comorbidities ^#^**	31 (5.2)	6 (7.1)	37 (5.5)
**Clinical outcome**			
Alive	589(99.5)	85 (100)	674 (99.6)
Dead	3 (0.5)	0	3 (0.4)

* *p* = 0.0086 and odds ratio = 0.44 for the proportions with symptomatic among vaccinated with two doses and vaccinated with one dose. ^#^ *p* = 0.0328 and Odds ratio = 0.49 for the proportions with individuals hospitalized with comorbidities and individuals hospitalized with non-comorbidities.

## Data Availability

All the sequencing data and information of this study is available in GISAID. Accession no is provided in supplementary table.

## Supplementary Materials

The following are available online at https://www.mdpi.com/article/10.3390/v13091782/s1, Table S1: Percentage genome retrieved, total reads mapped, and percentage relevant reads of the SARS-CoV-2 genomes retrieved in this study.
